# Clinical prediction model of Mycoplasma pneumoniae pneumonia combined with influenza virus infection in pediatric patients in Gansu, China

**DOI:** 10.1186/s12879-025-12498-7

**Published:** 2026-01-03

**Authors:** Jianzhi Zhang, Mei Peng, Yaxin Ma, Yonghong Sun

**Affiliations:** 1 The First Clinical Medical College, Gansu University of Chinese Medicine, Lanzhou, 730030 China; 2 Gansu Provincial Hospital, Lanzhou, 730030 China

**Keywords:** Influenza infection, Children, Mycoplasma pneumoniae, Prediction model, Community-acquired pneumonia, Novel inflammatory index

## Abstract

**Background:**

The incidence of respiratory infections in children has been increasing in recent years, and co-infections can lead to additional complications. This study aimed to investigate predictors of Mycoplasma pneumoniae pneumonia (MPP) co-infected with influenza virus through a retrospective analysis of clinical data in pediatric patients.

**Methods:**

We retrospectively reviewed the medical records of 195 children diagnosed with MPP at the Pediatric Internal Medicine Department of Gansu Provincial Hospital between November 2023 and November 2024. Patients were categorized into two groups: single-infection (*n* = 128, MPP alone) and mixed-infection (*n* = 67, MPP co-infected with influenza). Predictors of mixed infection were identified using a multivariate logistic regression-based prediction model. The model’s discrimination, accuracy, clinical utility, and generalizability were evaluated using receiver operating characteristic (ROC) curve analysis and decision curve analysis (DCA).

**Results:**

Multivariate analysis showed that influenza season, fibrinogen (Fib) level, fever duration, and C-reactive protein (CRP) were significantly associated with MPP co-infection (*p* < 0.05). The prediction model demonstrated good discrimination, with an area under the curve (AUC) of 0.820 (95% CI: 0.760–0.879) for the ROC analysis. DCA confirmed the model’s strong clinical utility.

**Conclusions:**

A prediction model based on influenza season, Fib level, fever duration, and CRP provide accurate identification of children at risk for MPP co-infected with influenza, demonstrating strong discrimination and clinical applicability.

**Clinical trial:**

Not applicable.

## Background

Mycoplasma pneumoniae (MP) is a common pathogen responsible for pediatric respiratory tract infections and a major cause of pediatric community-acquired pneumonia, referred to as Mycoplasma pneumoniae pneumonia (MPP) [1]. In recent years, the global incidence of pediatric infections caused by macrolide-resistant MP has been rising, with prevalence rates in China reported as high as 90% [2]. This increasing resistance poses substantial challenges for the clinical management of MPP in children. In addition, evidence shows that children co-infected with MP and other pathogens—notably influenza virus and adenovirus—have a higher risk of developing refractory Mycoplasma pneumoniae pneumonia (RMPP) than those with MP infection alone [3, 4]. However, clinical signs and symptoms alone are insufficient to reliably identify viral co-infections or mixed infections, since many respiratory pathogens (including bacteria and mycoplasma) can produce influenza-like presentations that mimic true influenza infection [5, 6]. Influenza itself remains a major cause of acute respiratory illness in children, accounting for over 20% of pediatric infections annually and contributing to roughly 650,000 deaths worldwide each year [7]. Therefore, early and accurate recognition of MP co-infection with influenza is clinically important for guiding timely management and improving outcomes in pediatric MPP. In this retrospective study, we analyzed clinical data to identify predictors of MP combined with influenza infection, with the aim of facilitating earlier detection and better patient prognoses.

## Methods

### Participants

Clinical data from 128 pediatric patients with single MP infection and 67 patients with MP co-infected with influenza virus treated at the Pediatric Internal Medicine Department of Gansu Provincial Hospital from November 2023 to November 2024 were retrospectively reviewed. The study was approved by the Hospital Ethics Committee (Approval No. 2024 − 653).

The sample size was determined according to the principle that the number of cases should be 10–20 times the number of predictive variables to ensure stable parameter estimation [8, 9]. Considering the limited number of positive events and the clinical difficulty of recruiting pediatric patients with MPP and influenza co-infection, the sample size was deemed acceptable for model construction.

Inclusion criteria: (1) Children meeting disease-specific diagnostic criteria, defined as follows: (a) For community-acquired pneumonia (CAP): the Consensus Document on Community-Acquired Pneumonia in Children jointly issued by the Spanish Society of Pediatric Pulmonology, the Spanish Society of Pulmonology and Thoracic Surgery, and the Spanish Society of Pediatric Infectious Diseases (SENP-SEPAR-SEIP) [10]; (b) For macrolide-resistant Mycoplasma pneumoniae pneumonia: the Antimicrobial Therapy of Macrolide-Resistant Mycoplasma Pneumoniae Pneumonia in Children [11]; and (c) For seasonal influenza: the Seasonal Influenza in Adults and Children–Diagnosis, Treatment, Chemoprophylaxis, and Institutional Outbreak Management: Clinical Practice Guidelines of the Infectious Diseases Society of America [12]. (2) Age between 28 days and 14 years, regardless of gender; (3) Complete and reliable medical records.

Exclusion criteria: (1) Children with obesity, hyperlipidemia, malnutrition, chronic diarrhea, or other chronic metabolic disorders; (2) Children with a history of chronic cor pulmonale, immune system disorders, or other clinically significant diseases affecting major organs; (3) Children with intellectual disabilities or psychiatric disorders; (4) Children previously treated with hormones, immunosuppressants, or other medications affecting immune function or metabolism; (5) Children missing key laboratory data due to refusal of tests upon admission; (6) Children co-infected with bacteria or other viruses.

### Measures

#### Etiological detection

Throat swabs were collected from children upon admission for pathogen detection. Influenza A and B viruses were detected using immunofluorescence assays. Additionally, reverse transcription-polymerase chain reaction (RT-PCR) was conducted using standardized diagnostic kits (CapitalBio Corporation, Beijing, China) to detect viral nucleic acids, MP, and Chlamydia pneumoniae.

#### Hematological tests

Fasting venous blood samples were collected at admission and analyzed in accordance with the manufacturer’s protocols for each specific testing kit. Laboratory indicators included C-reactive protein (CRP), procalcitonin (PCT), lactate dehydrogenase (LDH), albumin (ALB), aspartate aminotransferase/alanine aminotransferase ratio (AST/ALT), D-dimer (D-Di), fibrinogen (Fib), neutrophil (NEUT) percentage, platelet (PLT) count, lymphocyte (LY) percentage, creatine kinase-MB (CK-MB), interleukin-6 (IL-6), and serum amyloid A (SAA).

#### Data collection

Clinical and laboratory data were extracted and verified from the electronic medical record system of Gansu Provincial Hospital. Collected variables included demographic data (age, sex), clinical symptoms (cough, sputum production, wheezing, fever, duration of fever, peak body temperature), laboratory findings (as listed above), imaging results (location and number of pulmonary consolidations), hospitalization duration, pneumonia classification, and whether alveolar lavage was performed. All variables were coded and reviewed by two independent researchers to ensure accuracy and consistency.

### Statistical analysis

Prior to analysis, the dataset was systematically cleaned and validated to ensure accuracy and completeness. Records with inconsistent or missing entries were cross-checked against the original medical records by two independent reviewers, and any discrepancies were resolved through consensus, consulting attending physicians or verifying laboratory reports as needed.

Categorical variables were summarized as counts and percentages [n (%)] and compared using the chi-square (χ²) test. The normality of continuous variables was assessed using Quantile-Quantile (Q-Q) plots in combination with the Kolmogorov-Smirnov (K-S) test. Variables conforming to, or approximately following, a normal distribution were presented as mean with standard deviation [*M* (*SD*)], and differences between groups were evaluated using the independent-samples *t*-test. Non-normally distributed variables were expressed as median with interquartile range [*Mdn* (*IQR*)], and group comparisons were conducted using the Mann-Whitney *U* test.

Univariate analysis was first conducted to identify potential risk factors for Mycoplasma pneumoniae pneumonia co-infected with influenza. Variables of clinical relevance without evidence of multicollinearity were further screened using the generalized variance inflation factor (GVIF). Variables significantly associated with mixed infection in univariate analysis were entered into a multivariate logistic regression model using backward stepwise selection. Continuous predictors were categorized based on clinically meaningful cutoffs or thresholds derived from receiver operating characteristic (ROC) curve analysis to optimize discrimination.

Significant variables were entered into a multivariate logistic regression model using backward stepwise selection. The predictive performance of the final model was evaluated using ROC curves and the corresponding area under the curve (AUC) values. A nomogram was constructed to facilitate clinical interpretation and application. Decision curve analysis (DCA) was performed to assess the net clinical benefit of the model across a range of threshold probabilities.

Bootstrap resampling with 1,000 iterations was employed to internally validate the model and evaluate its stability, providing robust estimates of model performance and correcting for potential overfitting [13, 14]. All statistical analyses were conducted using R software (version 4.4.2).

## Results

### Clinical characteristics

A total of 195 children were enrolled in this study, including 128 cases in the single-infection group and 67 cases in the mixed-infection group. Compared to the single-infection group, the mixed-infection group had significantly higher D-dimer (D-Di) levels and platelet-to-lymphocyte ratio (PLR)-to-normal value ratio (*p* < 0.05), and significantly lower rates of Mycoplasma pneumoniae (MP) 23 S rRNA A2063/2064G mutation (*p* < 0.05) (see Table 1).

**Table 1:** General information and clinical characteristics of children in the simple infection group and mixed infection group

Variables		Simple infection group (*n* = 128)	Mixed infection group (*n* = 67)	*p*-value
Demographics				
	Age (years) [*Mdn* (*IQR*)]	7.00 (5.00–9.25)	7.00 (4.00–9.00)	0.505
	Sex (male) [*n* (%)]	52 (40.6)	31 (46.3)	0.546
Clinical symptoms				
	Productive cough [*n* (%)]	112 (87.5)	54 (80.6)	0.283
	Wheezing [*n* (%)]	6 (4.7)	0 (0.0)	0.173
	Fever course (days) [*Mdn* (*IQR*)]	4.00 (3.00–6.00)	4.00 (3.00–6.00)	0.126
	Fever peak (℃) [*Mdn* (*IQR*)]	39.00 (38.00–39.40)	39.00 (38.50–39.50)	0.058
Laboratory examinations				
	NE (10⁹/L) [*Mdn* (*IQR*)]	4.33 (3.44–5.65)	4.41 (2.83–6.11)	0.422
	LYM (10⁹/L) [*Mdn* (*IQR*)]	2.04 (1.51–2.40)	1.83 (1.31–2.38)	0.163
	PLT (10⁹/L) [*Mdn* (*IQR*)]	248.00 (208.00–291.75)	248.00 (208.00–291.75)	0.938
	SAA (mg/L) [*Mdn* (*IQR*)]	48.26 (13.27–100.08)	31.13 (10.21–76.56)	0.156
	CRP (mg/L) [*Mdn* (*IQR*)]	10.00 (10.00–16.69)	10.00 (10.00–15.11)	0.765
	PCT (ng/mL) [*Mdn* (*IQR*)]	0.08 (0.05–0.13)	0.08 (0.05–0.17)	0.425
	IL-6 (pg/mL) [*Mdn* (*IQR*)]	15.03 (9.56–22.52)	13.67 (7.68–20.69)	0.664
	D-Di (µg/L) [*M* (*SD*)]	0.55 (0.41)	0.80 (1.13)	0.026
	Fib (g/L) [*M* (*SD*)]	3.94 (0.54)	3.77 (0.99)	0.109
	Alb (g/L) [*M* (*SD*)]	40.72 (2.74)	41.01 (3.43)	0.523
	AST/ALT [*M* (*SD*)]	2.38 (1.01)	2.35 (0.84)	0.851
	LDH (U/L) [*Mdn* (*IQR*)]	287.37 (257.89–324.00)	293.00 (263.44–335.56)	0.477
	CK-MB (U/L) [*Mdn* (*IQR*)]	17.03 (13.85–23.23)	19.54 (14.16–25.55)	0.174
	NLR-to-normal value ratio [*Mdn* (*IQR*)]	0.04 (0.00–0.73)	0.50 (0.00–1.90)	0.156
	PLR-to-normal value ratio [*Mdn* (*IQR*)]	80.78 (60.14–115.62)	104.44 (60.99–150.55)	0.032
	SII [*Mdn* (*IQR*)]	525.27 (358.60–736.37)	650.76 (332.14–1043.34)	0.169
	MP 23 S rRNA A2063/2064G mutation [*n* (%)]	112 (87.5)	49 (73.1)	0.021

Note. *Mdn* = median, *IQR* = interquartile ranges, *M* = mean, *SD* = standard deviation

NE = neutrophils, LYM = lymphocytes, PLT = platelets, SAA = serum amyloid A, CRP = C-reactive protein, PCT = procalcitonin, IL-6 = interleukin-6, D-Di = D-dimer, Fib = fibrinogen, Alb = albumin, AST = aspartate aminotransferase, ALT = alanine aminotransferase, LDH = lactate dehydrogenase, CK-MB = creatine kinase-MB, NLR = neutrophil-to-lymphocyte ratio, PLR = platelet-to-lymphocyte ratio, SII = systemic immune-inflammation index, MP = mycoplasma pneumoniae

NLR normal ranges: 0–1 year 0.5–1.5, 1–3 year 0.7–1.7, 3–10 year 1.0–2.0, 10–18 year 1.0–2.5 [15]

PLR normal ranges: 0–1 year 10–30, 1–3 year 12–35, 3–10 year 15–40, 10–18 year 13–43 [15]

No statistically significant differences were found regarding gender, age, clinical manifestations, or novel inflammatory indices such as systemic immune-inflammation index (SII) and neutrophil-to-lymphocyte ratio (NLR)-to-normal value ratio, as well as consolidation site, between the two groups (*p* > 0.05) (see Table 1).

### Multivariate logistic regression analysis

**Fig. 1:**
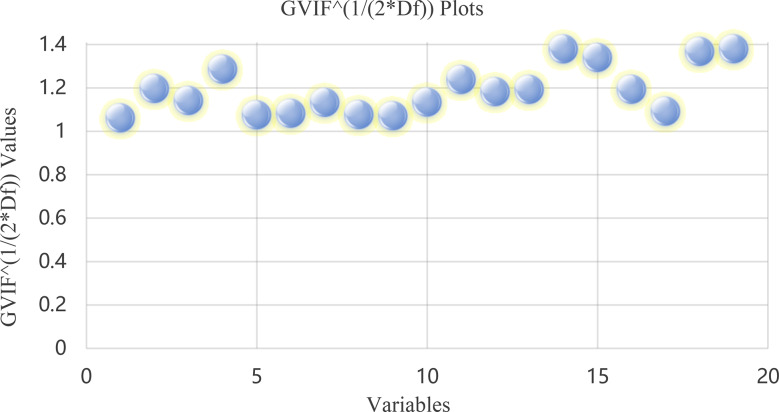
Generalized variance inflation factor (GVIF) plot adjusted for degrees of freedom. *Note*. Each point represents a predictor variable included in the multivariate logistic regression model: (1) Sex, (2) Disease course before admission, (3) Season, (4) Fever course, (5) Fever peak, (6) Cesarean delivery, (7) Premature infant, (8) Wheezing, (9) Systemic immune-inflammation index (SII), (10) Number of lung lobes involved in consolidation, (11) C-reactive protein (CRP), (12) Procalcitonin (PCT), (13) D-dimer(D-Di), (14) Fibrinogen (Fib), (15) Albumin (Alb), (16) Aspartate aminotransferase/alanine aminotransferase (AST/ALT) ratio, (17) Creatine kinase-MB (CK-MB), (18) Neutrophil-to-lymphocyte ratio (NLR)-to-normal value ratio, and (19) Platelet-to-lymphocyte ratio (PLR)-to-normal value ratio. All variables showed GVIF values close to 1, indicating no significant multicollinearity

Variables with GVIF values below 1.41 were selected to avoid multicollinearity (see Fig. 1). Multivariate logistic regression analysis indicated that influenza season, Fib, fever course, and CRP levels were significantly associated with mixed influenza virus infection in pediatric MPP patients (see Table 2).

**Table 2:** Multivariate logistic regression analysis of predictors

Variables	OR (95% CI)	*p*-value
Flu season	15.08 (6.423–40.560)	< 0.001
Fib	0.33 (0.174–0.575)	< 0.001
Fever course	1.21 (1.039–1.412)	0.015
CRP	1.03 (1.006–1.059)	0.023
SII	2.10 (0.961–4.728)	0.780

Note. OR = odds ratio; CI = confidence interval; Fib = fibrinogen; CRP = C-reactive protein; SII = systemic immune-inflammation index

Fever course = duration of fever (days) before hospital admission

Specifically, influenza season markedly increased the odds of co-infection (OR = 15.08, 95% CI: 6.42–40.56, *p* < 0.001), whereas higher Fib levels were associated with reduced odds (OR = 0.33, 95% CI: 0.17–0.58, *p* < 0.001). Longer fever duration (OR = 1.21, 95% CI: 1.04–1.41, *p* = 0.015) and elevated CRP (OR = 1.03, 95% CI: 1.01–1.06, *p* = 0.023) were also significant predictors. The SII did not show a statistically significant association with co-infection (OR = 2.10, 95% CI: 0.96–4.73, *p* = 0.78) (see Table 2).

### Evaluation of the predictive model for MPP combined with influenza virus infection

**Fig. 2:**
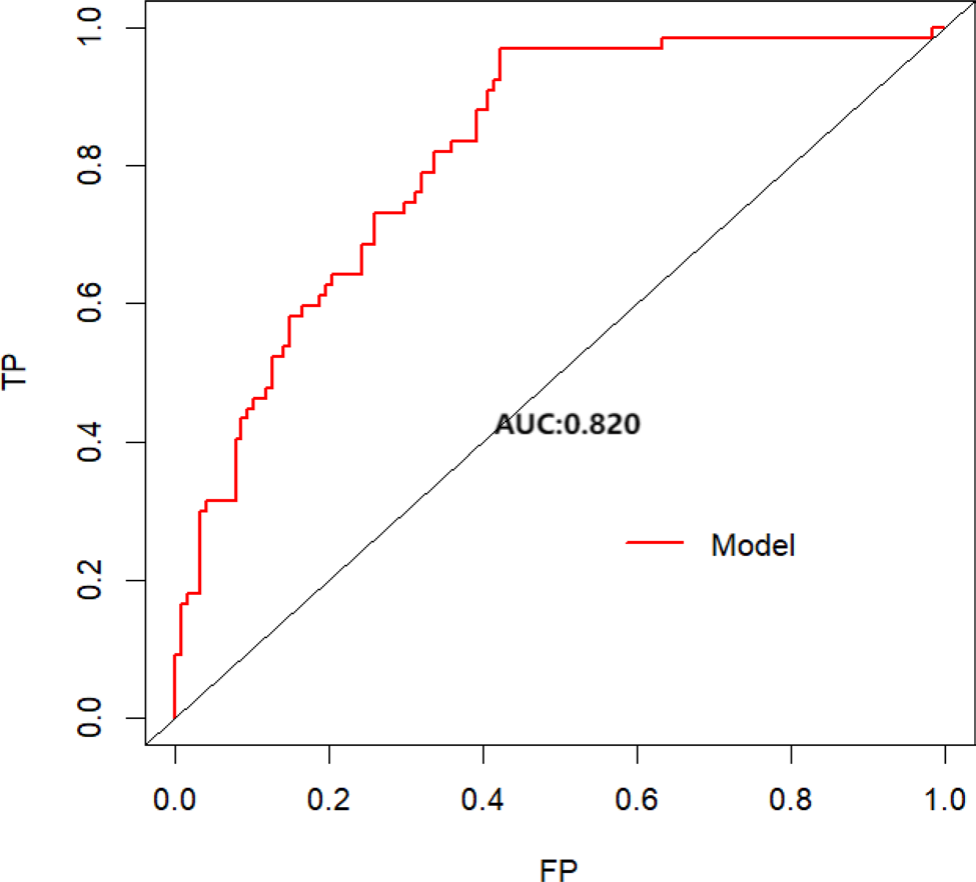
The area under the ROC curve of the model. *Note*. TP = True positive, FP = False positive, AUC = Area under the curve

The predictive model was evaluated using ROC analysis, which quantifies the model’s ability to distinguish between children with MPP alone and those with MPP co-infected with influenza. The model achieved an AUC of 0.820 (95% CI: 0.760–0.879) (see Fig. 2), indicating good discriminative performance, as AUC values between 0.8 and 0.9 are generally considered clinically useful. At the Youden-optimized threshold of 0.164, the model demonstrated high sensitivity (97.0%), meaning it correctly identified nearly all true co-infections, and moderate specificity (57.8%), comparable to rapid influenza diagnostic tests (RIDTs) during early epidemic phases. The negative predictive value (NPV) of 97.4% indicates a low probability of missed co-infections, supporting the model’s use as a rule-out tool that could reduce unnecessary oseltamivir use by approximately 43%.

Bootstrap validation with 1,000 resamples, following established simulation protocols [13, 14], confirmed the stability of the predictive model. The mean AUC was 0.79 (95% CI: 0.70–0.88), with low variability (coefficient of variation = 5.41%), indicating consistent performance across simulated datasets. These results demonstrate that the model is robust, clinically applicable, and reported in accordance with the Transparent Reporting of a multivariable prediction model for Individual Prognosis or Diagnosis (TRIPOD) guidelines [16], ensuring transparency and reproducibility.

**Fig. 3:**
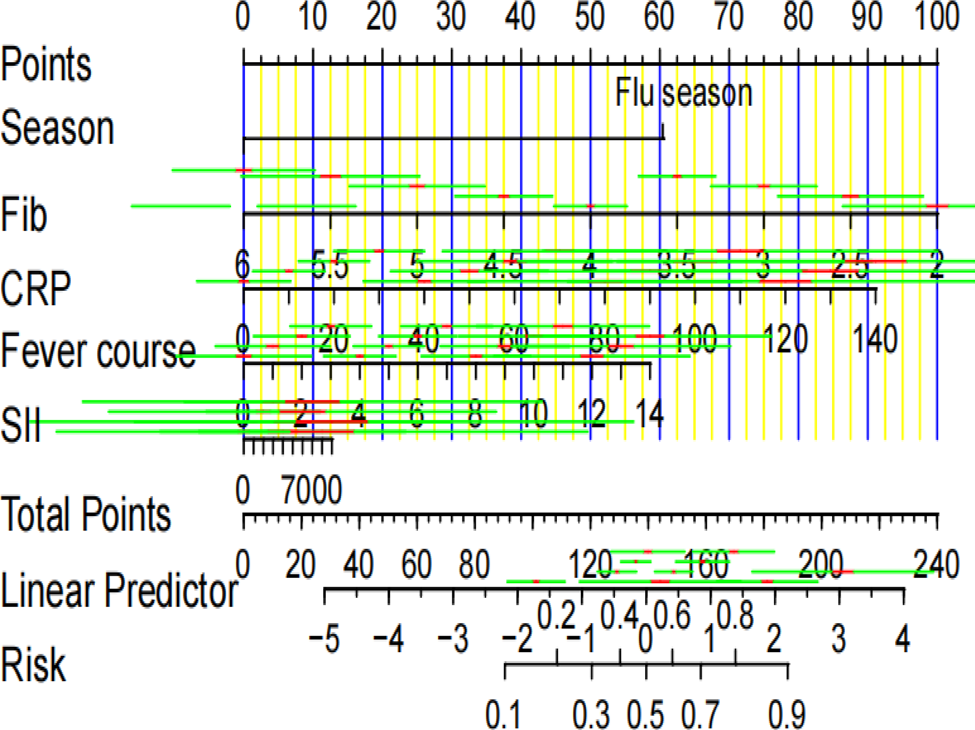
Nomogram of the model. *Note*. Fib = fibrinogen, CRP = C-reactive protein, SII = systemic immune-inflammation index. The red and yellow lines indicate the 95% confidence intervals

An alignment diagram-based prediction model was constructed to visualize the combined risk of influenza infection in children with MPP based on influenza season, Fib, fever duration, and CRP (see Fig. 3). For example, for a child during the influenza season with Fib = 4 g/L, fever duration = 4 days, and CRP = 60 mg/L, each risk factor corresponds to 60, 50, 16, and 40 points on the scoring scale, respectively. The total score of 166 translates to a predicted probability of approximately 75% for mixed influenza infection, illustrating practical application in individual risk assessment.

**Fig. 4:**
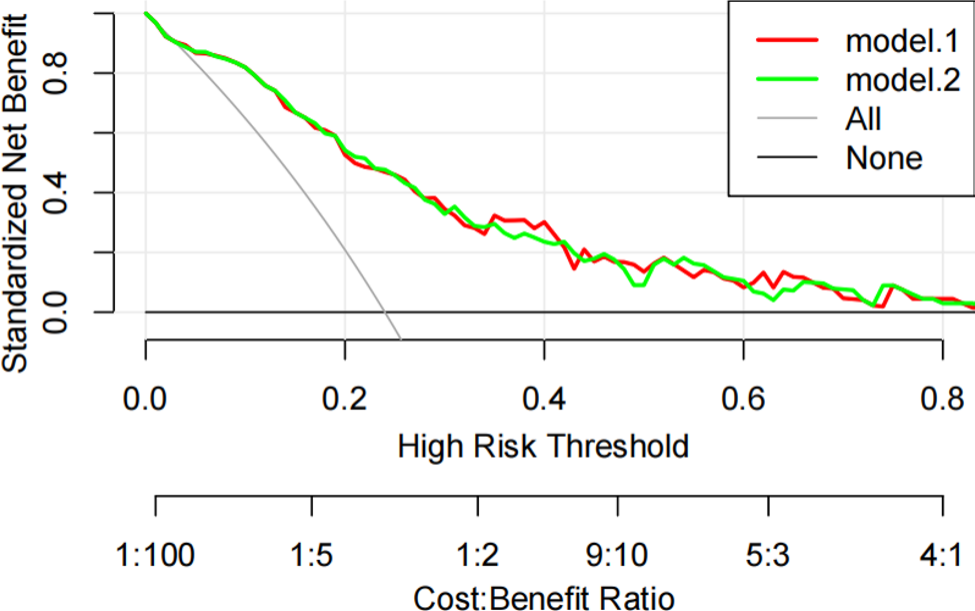
Clinical decision curve analysis. *Note*. Model.2 represents the original model, while Model.1 replaces SII with the PLR-to-normal value ratio. The black horizontal line indicates a net benefit of zero when no children receive the intervention. The gray diagonal line represents the net benefit if all children receive the intervention. The red and green curves show the net benefit of each model across different risk thresholds. SII = systemic immune-inflammation index, PLR = platelet-to-lymphocyte ratio

DCA evaluated the clinical benefit of the model (see Fig. 4). Across a threshold probability range of 3%–93%, including the additional univariate variable PLR-to-normal value ratio did not substantially improve the AUC compared to the original model (Model 1). This indicates that the model provides a net clinical benefit over traditional decision-making approaches, particularly when the diagnostic probability exceeds the minimal intervention threshold set by clinicians based on benefit-risk considerations.

## Discussion

In the current study, we developed and validated a predictive model for pediatric co‑infection of Mycoplasma pneumoniae (MP) and influenza virus. The model achieved an AUC of 0.82, a sensitivity of 97.0% and a NPV of 97.4%, indicating strong ability to identify children at high risk of mixed infection early. These results suggest that the model may help clinicians identify possible influenza co-infection among children already diagnosed with MP pneumonia, thereby supporting timely initiation of antiviral therapy and avoiding unnecessary empiric antibiotic escalation that is often triggered by uncertain co-infection status.

Our findings align with previous literature showing that viral co-infections in children with MPP are common and associated with more severe disease. For example, Yu et al. reported a viral co-infection rate of 38.8% among 748 children, with co-infected cases experiencing longer fever duration, higher Fib levels, and more complications [17]. Another multicenter investigation found multi-pathogen co-detection in almost 50% of MPP patients and significantly higher IL-6/IL-10 levels in co-infected compared to MP alone cases [18]. These data support the rationale for including fever duration, CRP, seasonal influenza activity and Fib in our predictive model.

Mechanistically, influenza co-infection is known to amplify cytokine release (e.g., IL-6) [19], resulting in increased CRP production [20, 21] and prolonged fever. Our observation that fibrinogen may act as a protective factor is plausible: acute inflammation typically increases fibrinogen synthesis, but dual infection may impair hepatocyte protein production or plasminogen activation, reducing Fib levels. Supporting this, studies on coagulation markers in children with MP found higher D-dimer and fibrinogen-degeneration-product levels in severe cases, suggesting coagulation dysfunction is linked to worse outcomes [22]. We also explored novel inflammatory indices such as the platelet-to-lymphocyte ratio (PLR) and the systemic immune-inflammation index (SII). Although SII has shown high AUC in predicting severity of MP pneumonia (AUC = 0.94) in other studies [23], in our cohort these indices added little net clinical benefit beyond our simpler model variables—suggesting that in the specific context of MP-influenza co-infection, more complex biomarkers may not substantially improve predictive utility. Bootstrap validation using 1000 resamples confirmed model stability, and adherence to the TRIPOD guidelines ensures transparency and reproducibility [16].

Importantly, these findings not only validate the predictive performance of the model but also highlight its potential real-world applicability: by integrating routinely available clinical parameters—season, Fib, fever duration, and CRP—clinicians can stratify pediatric patients by risk of MP-influenza co-infection, enabling early targeted interventions, rationalizing antibiotic and antiviral drug use, and optimizing resource allocation during influenza seasons.

Several limitations should be acknowledged. First, the retrospective, single‑center design may introduce selection bias and limits the generalisability of our findings to broader pediatric populations [4, 24]. Second, external validation of the prediction model was not conducted due to the relatively small number of pediatric patients with MPP and influenza co‑infection; future studies with larger, multicenter cohorts are needed to confirm the model’s robustness and applicability [17]. Third, the model’s specificity (~ 57.8%) is moderate, implying that false positives may occur, so clinical judgement remains essential when interpreting predictions. Fourth, mildly symptomatic children not requiring hospitalization were excluded, potentially limiting the applicability of the model to less severe cases. Fifth, some potentially relevant risk factors—such as prior vaccination status, comorbidities, or environmental exposures—were not included in the current dataset, which may affect predictive accuracy. Sixth, the detection of microorganisms in throat swabs cannot fully distinguish true infection from simple colonization, which may introduce misclassification bias.

Future research should prospectively evaluate the model’s impact on clinical outcomes—such as hospital length-of-stay, complication rates, and antiviral or antibiotic use—and explore integration with rapid viral diagnostic tools, including metagenomic or targeted next-generation sequencing, to enhance timely identification of co-infections [25, 26].

## Conclusion

This study developed a predictive model incorporating season, Fib, duration of fever, and CRP levels to assess the risk of pediatric MPP co-infected with influenza virus. The model demonstrated good discriminative ability, enabling early identification of high-risk children and supporting timely, targeted clinical interventions. Key limitations include the retrospective, single-center design, relatively small sample size, exclusion of mildly symptomatic children, and omission of potentially relevant risk factors such as prior vaccination status, comorbidities, or environmental exposures, which may limit generalizability. Future multicenter, prospective studies with larger cohorts are needed to validate the model, explore additional predictive factors, and assess its impact on clinical outcomes, antibiotic/antiviral use, and decision-making in real-world pediatric care.

## Data Availability

Due to ethical restrictions and the sensitive nature of the data, the datasets generated and/or analyzed during the current study are not publicly available. However, data are available from the corresponding author on reasonable request and with permission from the ethics committee at the institution.
